# Super refractory status in a case of Febrile Infection‐Related Epilepsy Syndrome due to hemophagocytic lymphocytic histiocytosis

**DOI:** 10.1002/epi4.12454

**Published:** 2021-01-15

**Authors:** Salvadeeswaran Meenakshi‐Sundaram, Muthukani Sankaranarayanan, Murugan Jeyaraman, Chitra Ayyappan, Somalinga Nagendran Karthik, Suresh Pandi

**Affiliations:** ^1^ Department of Neurosciences Apollo Speciality Hospitals Madurai India; ^2^ Department of Pediatrics Apollo Speciality Hospitals Madurai India

**Keywords:** anticonvulsant polytherapy, Febrile Infection‐Related Epilepsy Syndrome, hemophagocytic lymphocytic histiocytosis, immunomodulation, super refractory status epilepticus

## Abstract

A 14‐year‐old boy presented with a prodromal respiratory infection followed by super refractory status epilepticus. A diagnosis of Febrile Infection‐Related Epilepsy Syndrome (FIRES) was made. Initial MRI study and CSF analysis were normal. He required multiple anticonvulsants owing to the refractory nature of the seizures. The course of the illness was rather stormy, laced with various medical problems viz. hepatic dysfunction, sepsis, hemodynamic, and hematological abnormalities which posed several challenges in the management. Hemophagocytic lymphocytic histiocytosis (HLH) was identified as the etiology of the illness and was treated but without success. The case report highlights the several immunomodulatory strategies that were employed to treat the disease, despite which the outcome was unfavorable.

**Key Points**

- Febrile Infection‐Related Epilepsy Syndrome (FIRES) presents as a super refractory status epilepticus (SRSE).
- Management of FIRES may require multiple anticonvulsants and immunomodulatory strategies.
- Multiple medical problems could be faced during the management of such a patient, related to both the presentation ie FIRES and its etiology, namely hemophagocytic lymphocytic histiocytosis (HLH).
- Pursuit of etiology, as for instance HLH in this case, is important from the therapeutic point of view.
- Early and aggressive management is of paramount importance in this difficult‐to‐treat disorder.

## 1 INTRODUCTION

Febrile Infection‐Related Epilepsy Syndrome or FIRES is a potentially devastating disease characterized by refractory status epilepticus that occurs following an apparently innocuous febrile illness in previously healthy people, usually children of school‐going age.^1^ We report a patient with the syndrome in whom an unusual etiology was found, and the patient succumbed despite appropriate management strategies. We discuss here the potential difficulties faced during the management of the patient.

## 2 CASE REPORT

A 14‐year‐old boy was admitted with a seizure. He first experienced throat pain and rhinorrhea 9 days before this presentation and was treated with amoxicillin‐clavulanate. Seven days before this admission he started experiencing fever followed by headache and vomiting. Since six days, there was history of decline in food intake. He progressively worsened. A few hours before this admission, he experienced a seizure characterized by sudden alteration of sensorium followed by tonic posturing of neck, face, and hand with deviation of face to the right side. Seizure lasted less than a minute, and he remained in altered sensorium since. He had no comorbidities earlier. Birth, development, and immunization history were unremarkable and family history was negative for any similar or severe illnesses.

At admission, his vital parameters were as follows: pulse: 102/minute, BP: 110/80 mm Hg, respirations: 30 per minute, and temperature 98.4°F. Cardiorespiratory and per abdominal examinations were normal. Neurologically, he was drowsy and responding to pain. There was no word output. There were no meningeal signs or papilledema. Pupils were normal and reactive. Eye movements were normal. Stretch reflexes were normal, and plantars were flexors.

Levetiracetam (1.5 G stat, followed by 750 mg twice daily intravenously), acyclovir (10 mg/kg thrice a day intravenously), and supportive care measures were instituted.

Serum chemistry revealed transaminitis (aspartate aminotransferase, ie AST 466 i.u/L, alanine aminotransferase, ie ALT 360 i.u/L). Alkaline phosphatase was elevated (270 i.u/L). Bilirubin (0.8 mg%), renal functions, electrolytes, calcium, and phosphorous were normal. Hemogram revealed relative leucopenia (total WBC count 3700/cu.mm). ESR was 7 mm/hour. A contrast‐enhanced MRI of the brain was normal. He tested negative for malarial parasite, microfilaria, dengue, and leptospira. Initial blood and urine cultures were sterile. Widal test was negative. Lumbar puncture revealed clear CSF with normal opening pressure. CSF analysis revealed: total count: 5 cells, all lymphocytes, protein 62 mg%, and normal sugar. CSF virological studies were negative. Acyclovir was subsequently withdrawn.

On day 2, he experienced seizure again. Lacosamide (300 mg stat followed by 150 mg twice daily intravenously) was administered. Sensorium further worsened. Arterial blood gas analysis (ABG) revealed serial worsening with evolution of type II respiratory failure, warranting intubation, and ventilation. Serum ammonia was elevated (101 µmol/L, normal 12‐47 µmol/L). Levocarnitine was added. Continuous EEG monitoring was started and showed recurrent epileptiform activity arising from both fronto‐temporal regions, left more than right.

Subsequently, he started experiencing recurrent clinical seizures. Midazolam infusion (0.2 mg/kg stat followed by 0.5 mg/kg/hour infusion) was started. A drop in blood pressure warranted ionotropic support. He was now started on pulse methylprednisolone (1 g by intravenous infusion, once daily for 5 days). Hyperglycemia was noticed requiring administration of insulin. Seizures still recurred when ketamine infusion (3 mg/kg stat followed by 3 mg/kg/hour infusion) was started and burst suppression pattern was achieved.

On day 3, he was also started on intravenous immunoglobulin (IVIg) (2 g/kg administered over next five days). Ketogenic diet was instituted.

**FIGURE 1:**
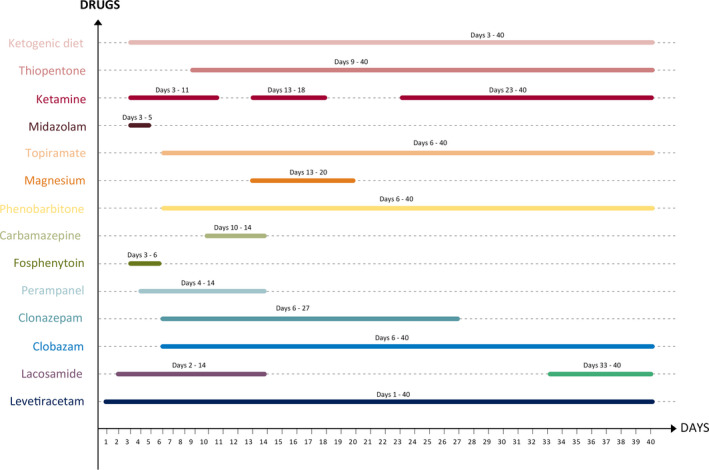
Figure showing the various anticonvulsants and the timelines

**FIGURE 2:**
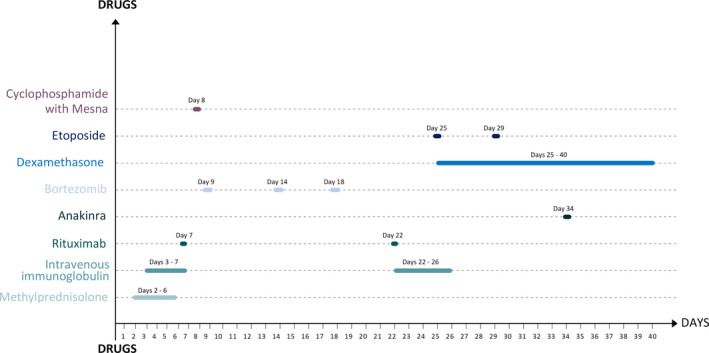
Figure showing the various immunomodulatory strategies and the timelines

Multiple anticonvulsants were needed during the course of the disease, titrated based on metabolic and hemodynamic parameters, and are summarized in Figure 1. The various immunomodulatory strategies are summarized in Figure 2.

Autoimmune workup revealed strongly positive anti‐nuclear antibodies on immunofluorescence with a homogenous nuclear pattern. Extractable nuclear antigen test was negative. Lupus anticoagulant test was weakly positive. Antithyroid antibody titers were strongly positive (serum antithyroid peroxidase‐O, ie TPO antibodies 112.23 IU/mL, normal: <5.61 IU/mL). All other tests of autoimmune panel including serum and CSF autoimmune antibody profile (anti NMDA, LGI‐1, CASPR‐2, GAD, GABA‐A, GABA‐B, AMPA antibodies in serum and CSF; serum anti‐ds‐DNA, beta‐2 glycoprotein, and cardiolipin antibodies, angiotensin‐converting enzyme levels, VDRL, p‐ANCA, and c‐ANCA, rheumatoid factor) were negative.

Bilirubin levels progressively increased (Table 1), predominantly the conjugated bilirubin levels. Ultrasound of the abdomen revealed mild ascites and bilateral pleural effusion. Repeat MRI of the brain showed T2/FLAIR hyperintensities in both mesial temporal lobes.

**TABLE 1:** showing the serial hematological and liver function parameters

Laboratory parameters	Day 1	Day 8	Day11	Day13	Day 16	Day 21	Day 33
Hemoglobin (g/dL)	13.6	11.4	10.9	7.5	10.2	9.1	7.4
Total leukocyte count (×10^9^/L)	3.7	7.8	2	5.7	23.7	9.4	2.9
Platelets (×10^9^/L)	220	300	200	100	50	160	130
Total bilirubin (mg/dL)	0.6	3.7	11.9	13.2	9.6	18.7	28.7
Direct bilirubin (mg/dL)	0.5	3.4	10.4	11.2	8.9	15.1	22.3
Aspartate amino transferase (AST) (U/L)	466	139	208	104	67	79	66
Alanine amino transferase (ALT) (U/L)	635	96	131	104	67	53	47
Alkaline phosphatase (IU/L)	270	198	670	337	263	330	263
Total Protein (g/dL)	6.5	6.2	6	4.7	4.8	5.9	5.6
Albumin (g/dL)	4.2	3.2	3.3	2.6	1.9	2.4	3

**FIGURE 3:**
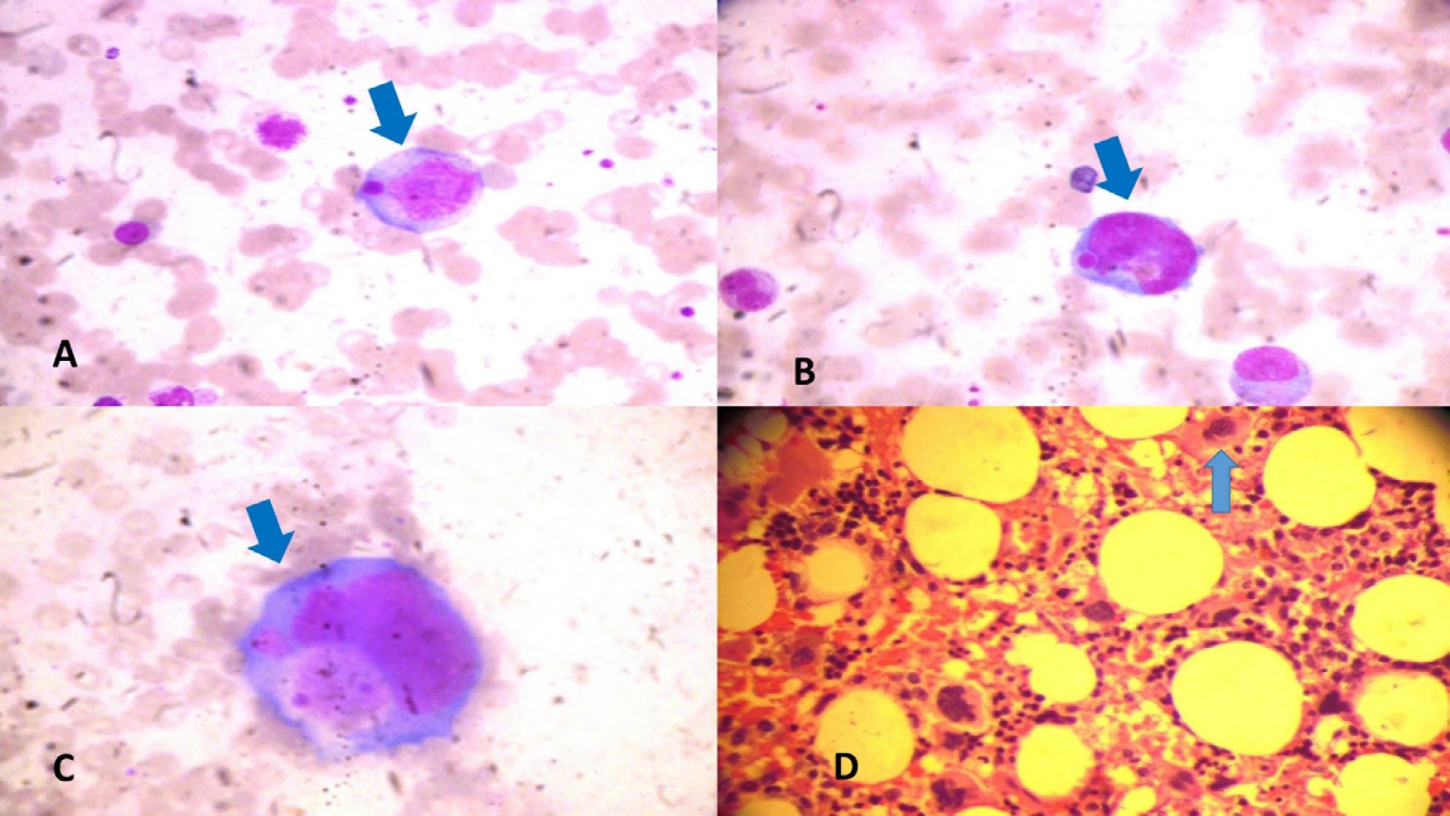
(A, B, C‐ Bone marrow aspiration, D‐ Bone marrow biopsy) shows phagocytosis of hematopoietic elements by macrophages (Hematoxylin‐Eosin stain) (A, B, C‐ 100*10 × magnification, D‐ 40*10 × magnification)

Serum ferritin levels were elevated on admission (2719 ng/mL, normal 7‐140 ng/mL) and progressively rose to 13 341 ng/mL on day 35. Serum triglyceride levels were elevated on admission (211 mg/dL, normal <150 mg/dL) and rose to 685 mg/dL on day 35. Bone marrow examination revealed features of hemophagocytic lymphocytic histiocytosis (HLH) with macrophage activate syndrome (Figure 3). Serum CD19 and 20 levels, soluble CD‐25, CXCL‐9, and IL‐18 levels, and NK cell function studies could not be tested. Whole‐genome exon sequencing did not reveal any evidence to suggest familial HLH.

Etoposide (150 mg/m^2^ intravenously, repeated twice weekly) and dexamethasone (10 mg/m^2^ intravenously daily) were administered. On day 34, owing to further recurrences of seizures, anakinra (100 mg subcutaneously) was started. He had multiple recurrences of seizures following this, and hence, anakinra was not continued.

He required multiple courses of antibiotics during the stay, administered based on culture‐sensitivity patterns on blood, urine, and endotracheal aspirate. He required administration of granulocyte‐monocyte colony‐stimulating factor (GM‐CSF) (175 micrograms subcutaneously) when the blood counts dropped after the immunomodulatory therapy (Table 1). He also received pneumocystis carinii prophylaxis (cotrimoxazole). He continued to experience multiple recurrences of seizures.

On day 40, he developed cardiorespiratory arrest and succumbed to the illness.

## 3 DISCUSSION

Our patient had super refractory status epilepticus on a background of recent febrile illness. Status epilepticus persisting despite two appropriately chosen anticonvulsants and including one benzodiazepine administered in therapeutic doses is considered as refractory in nature.^2^ Status epilepticus lasting more than 24 hours after initiation of anesthesia or recurring following withdrawal/ tapering of anesthesia is called as super refractory status epilepticus.^3^ Such a presentation occurring in a patient with no past history of epilepsy and without acute or active metabolic, toxic, or structural cause warrants consideration of the syndrome of NORSE ie new‐onset refractory status epilepticus.^4^ NORSE may or may not be preceded by fever and when fever precedes the onset of such seizures by 2 weeks to 24 hours, it is referred to as FIRES ie Febrile Infection‐Related Epilepsy Syndrome.^1^ Our patient fulfilled the consensus definitions of NORSE and FIRES. ^5^

Management of FIRES is challenging and can be classified into four categories: (i) general supportive measures, (ii) management of the seizures, (iii) immunotherapy, and (iv) Treatment of any identifiable etiology.

General supportive measures posed several challenges in our patient. He required prolonged ventilatory assistance with its attendant complications, importantly pneumonia, and required several courses of antibacterial and antifungal agents. Diarrhea associated with the use of antibiotics as well as ketogenic diet posed additional challenges. Maintenance of nutritional balance in the wake of need for ketogenic diet was particularly difficult. Additional measures included prevention of decubitus ulcers, prevention of skin excoriations despite the need for repeatedly cleaning the fecal‐soiled skin areas, especially in times of diarrhea, prevention of deep vein thrombosis, and passive physiotherapy measures. Parents and family members needed extensive counseling measures since the child required extended stay in the intensive care unit. In India, financial issues could pose additional challenges. Extensive evaluation, management in intensive care area, use of costly equipment like ventilator and infusion pumps, long‐term hospital stay, and the use of expensive medications like immunoglobulin, chemotherapeutic agents, and antibiotics can be demanding financially, especially in the absence of medical insurance.

The second pillar of management was anticonvulsants. It is well‐known that patients with FIRES respond poorly to anticonvulsants. Polytherapy is the rule as was seen in our patient. Levetiracetam was the first anticonvulsant used in our patient followed by lacosamide when he had a recurrence. When seizures subsequently recurred in clusters, FIRES was considered and midazolam was added and then ketamine. Abnormality of liver function tests in the form of anicteric transaminitis and hyperammonemia at admission and then the appearance and worsening of jaundice precluded the use of valproate. Anticipating the pharmacoresistant nature of the illness, we introduced ketogenic diet early in the course of the illness. Despite encouraging results reported in response to such a diet in literature, seizures were relentless in our patient.^6^ We used several conventional anticonvulsants viz. levetiracetam, lacosamide, fos‐phenytoin, clobazam, clonazepam, phenobarbitone, topiramate, and perampanel and additional agents namely midazolam, ketamine, thiopentone, and magnesium. We maintained prolonged burst suppression with ketamine and thiopentone, yet when attempts were made to taper off the same, even with minimal reductions in the doses, generalized seizures recurred.

The third aspect of management of FIRES is immunotherapy. We aggressively instituted early immunomodulation in the form of intravenous methylprednisolone and immunoglobulin. When seizures recur beyond a week, status epilepticus is said to be “prolonged,” and hence, we added further immunomodulation. ^7^ These were in the form of the anti‐CD 20 monoclonal antibody, rituximab, and the chemotherapeutic agent cyclophosphamide that has been anecdotally used in the past in the treatment of FIRES. We also used anakinra, a recombinant modified version of human interleukin‐1 receptor antagonist protein which could be useful in severe refractory autoimmune encephalitis.^8^ Peculiarly, our patient had a flurry of seizure upon administration of anakinra precluding further use of the same. Bortezomib is a 26‐S proteasome inhibitor used in the management of plasma cell dyscrasias such as multiple myeloma.^9^ Although some evidence exists regarding the role in refractory autoimmune encephalitis, it did not benefit our patient.^10^ A point of particular concern in our patient was the cytopenias that required additional therapy and also precluded timely administration of aggressive immunomodulatory therapy. Also, deranged liver functions precluded the use of tocilizumab in our patient.

The fourth aspect of management is the treatment of the etiology. The etiology of FIRES is identified in less than half of the patients and autoimmune and paraneoplastic causes are often implicated. ^11^ Autoimmune work up in our patient yielded positive anti‐nuclear and antithyroid antibodies. This warranted administration of conventional immunotherapy in the form of methylprednisolone and immunoglobulin. The patient had a progressive rise in bilirubin and also ferritin. Ferritin elevation warranted further evaluation when HLH was identified. Additional immunomodulation in the form of etoposide and dexamethasone were administered hence. ^12^ However, our patient did not respond to these measures and succumbed.

Hemophagocytic lymphocytic histiocytosis (HLH) is a rare life‐threatening hyperinflammatory hematological syndrome characterized by cytokine storm. ^13^ It can be primary or secondary to several conditions including autoimmune disorders, infections, and malignancy. The histiocytic society revised guidelines for the diagnosis of HLH, 2004 requires either molecular diagnosis consistent with HLH or the presence of five out of eight criteria, namely fever, splenomegaly, cytopenias, hyperferritinemia, hypertriglyceridemia or hypofibrinogenemia, hemophagocytosis in bone marrow or other tissue, increase in sCD25 or reduced/absent NK cell function. ^14^ Our patient had persistent fever, cytopenia, hyperferritinemia, hypertriglyceridemia, and bone marrow evidence of hemophagocytosis. Whether FIRES and HLH are the underpinnings of an underlying immune dysfunction in a genetically susceptible individual which manifests with cytokine storm following a febrile illness or one leading to another is not clearly known. Farias‐Moeller et al described 3 patients with FIRES and HLH and suggested abnormal activation of innate immune system following a febrile illness that leads onto release of cytokines and chemokines resulting in neuronal hyperexcitability and manifesting as refractory seizures. ^15^ The defective NK cell function in HLH leads onto defective clearance of antigen‐presenting cells that results in a vicious cycle with further activation of macrophages and monocytes followed by phagocytosis of hematopoietic elements and tissue damage. ^16^ Serum ferritin in our patient at the time of admission was elevated and further increased during the course of the illness suggesting that HLH preceded the onset of FIRES.

The case highlights the multiple problems that could be faced by clinicians in the management of this difficult‐to‐treat disorder that resulted in an unfavorable outcome.

## CONFLICT OF INTEREST

None of the authors has any conflict of interest to disclose. We confirm that we have read the Journal's position on issues involved in ethical publication and affirm that this report is consistent with those guidelines.
